# Real-time and accurate estimation of surgical hemoglobin loss using deep learning-based medical sponges image analysis

**DOI:** 10.1038/s41598-023-42572-6

**Published:** 2023-09-19

**Authors:** Kai Li, Zexin Cheng, Junjie Zeng, Ying Shu, Xiaobo He, Hui Peng, Yongbin Zheng

**Affiliations:** 1 Department of Gastrointestinal Surgery, Renmin Hospital of Wuhan University, Wuhan, Hubei China; 2 College of Informatics, Huazhong Agricultural University, Wuhan, Hubei China; 3 Department of Laboratory Medicine, Renmin Hospital of Wuhan University, Wuhan, Hubei China

**Keywords:** Surgery, Medical research, Biomedical engineering, Medical imaging

## Abstract

Real-time and accurate estimation of surgical hemoglobin (Hb) loss is essential for fluid resuscitation management and evaluation of surgical techniques. In this study, we aimed to explore a novel surgical Hb loss estimation method using deep learning-based medical sponges image analysis. Whole blood samples of pre-measured Hb concentration were collected, and normal saline was added to simulate varying levels of Hb concentration. These blood samples were distributed across blank medical sponges to generate blood-soaked sponges. Eight hundred fifty-one blood-soaked sponges representing a wide range of blood dilutions were randomly divided 7:3 into a training group (n = 595) and a testing group (n = 256). A deep learning model based on the YOLOv5 network was used as the target region extraction and detection, and the three models (Feature extraction technology, ResNet-50, and SE-ResNet50) were trained to predict surgical Hb loss. Mean absolute error (MAE), mean absolute percentage error (MAPE), coefficient (*R*^2^) value, and the Bland–Altman analysis were calculated to evaluate the predictive performance in the testing group. The deep learning model based on SE-ResNet50 could predict surgical Hb loss with the best performance (*R*^2^ = 0.99, MAE = 11.09 mg, MAPE = 8.6%) compared with other predictive models, and Bland–Altman analysis also showed a bias of 1.343 mg with narrow limits of agreement (− 29.81 to 32.5 mg) between predictive and actual Hb loss. The interactive interface was also designed to display the real-time prediction of surgical Hb loss more intuitively. Thus, it is feasible for real-time estimation of surgical Hb loss using deep learning-based medical sponges image analysis, which was helpful for clinical decisions and technical evaluation.

## Introduction

Real-time and accurate estimation of surgical blood loss plays a crucial role in fluid resuscitation management and evaluation of surgical techniques, which not only helps anesthesiologists perform perioperative patient management but also helps surgeons reduce the time of learning curve and improve the surgical techniques^1–3^. At present, perioperative evaluation of surgical blood loss mainly relies on the visual estimation of surgeons and anesthesiologists, which makes it highly subjective, inaccurate, and unreliable^4,5^. Although gravimetric analysis could also provide a measurement method of hemoglobin (Hb) by subtracting the known dry weight of laparotomy sponges from the blood-soaked sponges^6^, this method is inaccurate and time-consuming. After all, these blood-soaked sponges might be confounding non-sanguineous fluids and other substances, such as the ascites, saline, and other tissues, besides, the method also relies on the assumption that the Hb concentration of the intraoperative patient's blood is stable, which is an unreasonable assumption because the patient's blood from the intravenous infusion becomes increasingly diluted^7^. Several studies also showed the method for measuring Hb content from all blood-absorbing media, which is a more accurate procedure and has been described as a standard measurement method for surgical blood loss evaluation^8,9^. However, these methods are impractical and time-consuming for real-time intraoperative estimation. Therefore, the method for a real-time and accurate estimate of surgical Hb loss is urgently needed in clinical practice.

With the rapid development of artificial intelligence in clinical medicine over the past several decades, deep learning has emerged as a powerful technique for extracting feature information from medical images^10,11^, which has been widely applied in the field of image diagnosis and prediction with excellent performance owing to their advantages of being fast, accurate, and reproducible^12–14^. In the present study, we hypothesized that surgical Hb loss could be estimated using deep learning-based medical sponges image analysis, which could establish a determinate foundation for further clinical application. Therefore, the present study aimed to explore a novel surgical Hb loss estimation method using deep learning-based medical sponges image analysis, and evaluate and compare the performance of other predictive models.

## Methods

### Hemoglobin loss measurements

Whole blood samples were collected from the department of laboratory medicine, Renmin Hospital of Wuhan University, which were residual blood samples of the patient after routine blood analysis. Patients with diseases that would change the color of the blood were excluded from this study, such as hypoxia, carbon monoxide poisoning, nitrite poisoning, and jaundice. Patient Hb measurements were recorded, as reported in the patient’s medical record. The normal saline was added to simulate varying levels of hemodilution. The Hb concentration was selected, ranging from 50 to 170 g/L, which could represent the clinical range of Hb values^15^. The different blood samples with pre-measured Hb concentration were distributed across medical sponges by pipette to generate blood-soaked sponges. Then, the image with blood-soaked sponges was captured by the digital camera with the same height and parameters (Canon, EOS 200D II, Japan). The images were randomly divided 7:3 into training and testing groups, the images from the training group were used to develop the predictive model, and the images from the testing group were used to evaluate the model performance. Considering the small sample size, no validation set was carried out in this study, but we performed a five-fold cross validation experiment based on the training dataset to potentially minimize overfitting on the test data. Informed consent was obtained from each patient. This study was approved by the institutional review board of the Renmin Hospital of Wuhan University, in accordance with the 1964 Helsinki declaration and its later amendments or comparable ethical standards.

### Image processing

Since the tone of the sponges and background regions are closer, traditional image processing methods could not provide the accurate contour region of blood-soaked sponges. To effectively remove unnecessary background information, a deep learning model based on the YOLOv5 network was automatically used as the target region extraction and detection. The main work includes: (1) The sponge's area was precisely annotated by Labelmg-1.8.3 image annotation software, and stored as a .xml file, (2) the YOLOv5 network model was developed^16^. (3) Save the detected target area as input to the surgical Hb loss predictive network. In the YOLOv5 network, the image input size is 640 × 640 × 3, the Batch Size is set to 16, the initial learning rate is set to 1e−3, the total number of training iterations is set to 1000 Epoch, and the loss function is a mean squared error (MSE). The operating system was Ubuntu 16.04.7, and the main working platform was equipped with NVIDIA Tesla P100 PCle 16 GB GPUs. PyTorch was used to construct the deep learning network, and PyCharm was an integrated development environment applied for was used as the programming language in Python.

### Predictive models

Three predictive models were used to obtain the best surgical Hb loss prediction and evaluate the predictive performance, which included feature extraction technology, ResNet-50, and ResNet50-based squeeze-and-excitation module (SE-ResNet50). Besides, the optimal model was selected to design an interactive interface for model visualization. The system is divided into the front-end user interface (UI), presentation layer, business layer, data layer, database, and runtime environment, in which the front-end UI and presentation layer are implemented using the Vue framework, and the business layer and data layer are implemented using Flask structure, and depend on the database and the underlying runtime environment. In this framework, the image preprocessing and prediction model is placed in the back-end part, which preprocesses the region segmentation of the images received from the front-end part and then performs the prediction. The whole interactive interface realizes the functions, such as login, registration, single image prediction, total statistics of multiple images, etc., and stores the prediction data in the MySql database in real-time.

#### Feature extraction technology

**Figure 1:**
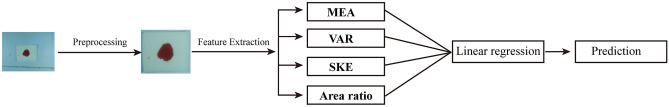
The linear regression models based on feature extraction technology.

Color moments have been proven to be effective in representing the color distribution in the image, which includes first-order moments (mean, MEA), second-order moments (variance, VAR), and third-order moments (skewness, SKE)^17,18^. MEA reflects the overall brightness of the image, VAR reflects the color distribution range of the image, and SKE reflects the symmetry of image color distribution. In the present, we extracted feature parameters from the image using feature extraction technology, such as MEA, VAR, SKE, and the area ratio of blood area to the sponge area (Fig. 1). These feature parameters were included in a linear regression model to predict surgical Hb loss, the final equations for MEA (X1), VAR (X2), SKE (X3), and the area ratio of blood area to the sponge area (X4) were as follows: $\hat{y} = b0 + b1X1 + b2X2 + b3X3 + b4X4$.

#### Deep residual network

**Figure 2:**
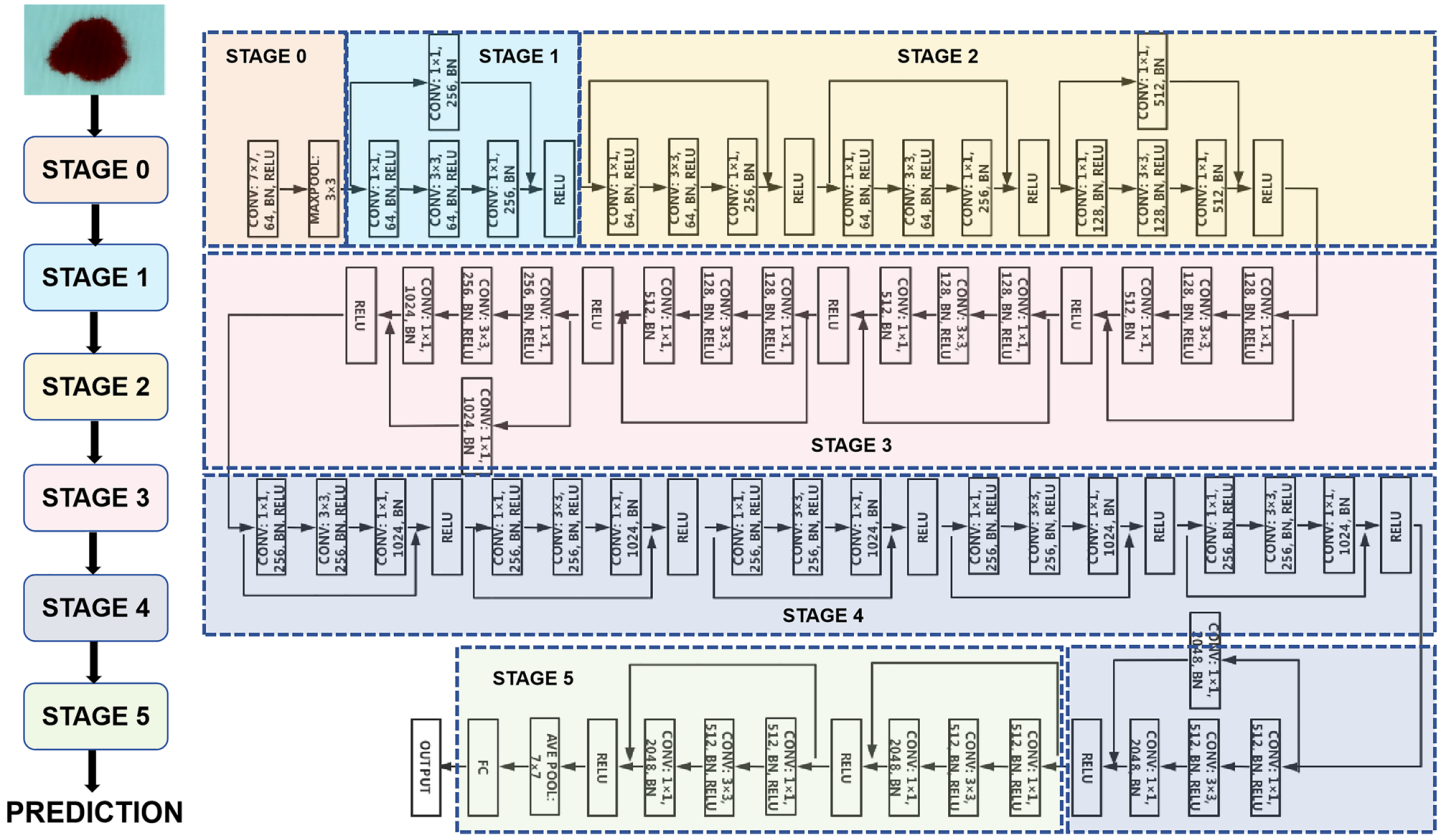
The model structure of the ResNet-50.

The residual network (ResNet) is a representative deep convolutional neural network widely used in the field of target classification, which can automatically learn the main features related to the target task in the image through gradient descent, relying on the effective feature extraction ability^19–21^. In the present study, we also constructed a deep learning model to predict surgical Hb loss based on the 50-layer residual network (ResNet-50), and the model structure is shown in Fig. 2. In the ResNet50 network, the image input size is 224 × 224 × 3, the Batch Size is set to 16, the initial learning rate is set to 1e−3, the total number of training times is set to 1000 Epoch, the loss function is MSE and L1 loss, and the optimizer is selected as Adam.

#### SE-ResNet50 network

**Figure 3:**
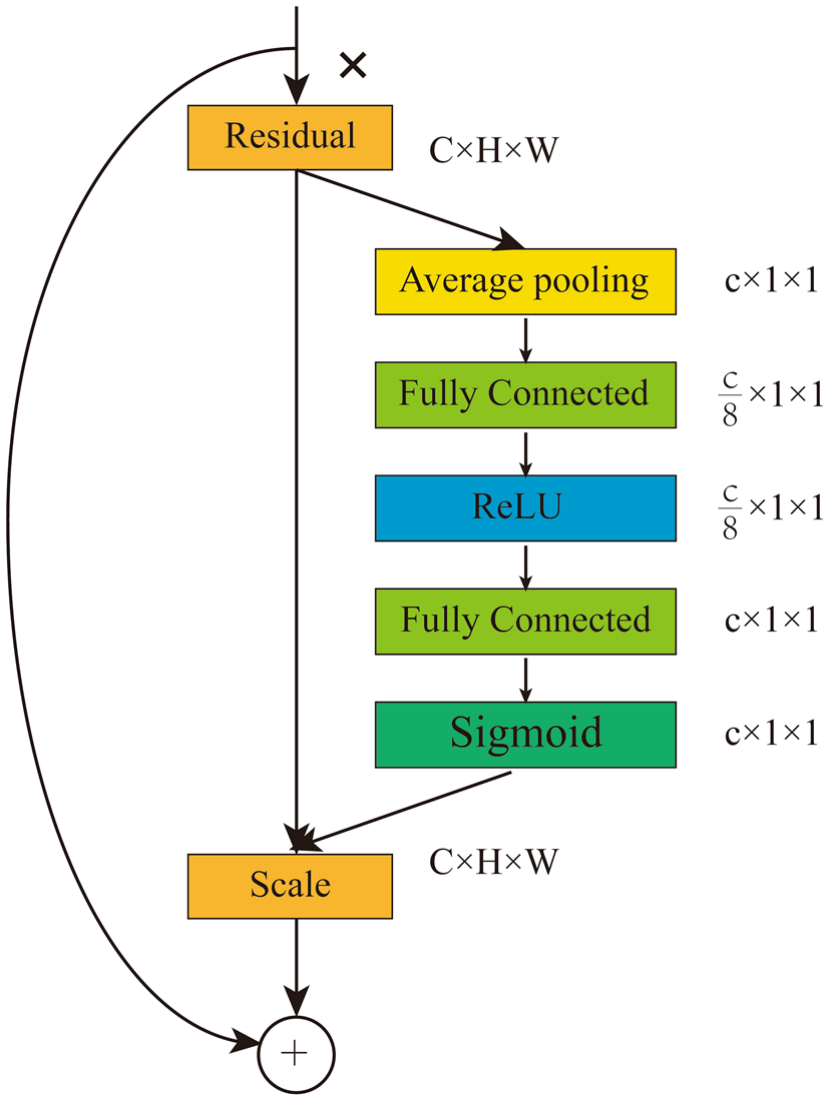
SE-ResNet50 Module.

SE-ResNet is the network in which squeeze-and-excitation (SE) blocks are added to the backbone network ResNet^22,23^. In the present study, we also constructed a deep learning model based on the SE-ResNet50 network to predict surgical Hb loss, which can not only further improve the ability to extract features and avoid excessive parameter loss of the module but also strengthen the network learning performance^24^. SE blocks after adding ResNet-50 are shown in Fig. 3. In the SE-ResNet50 network, the fundamental hyperparameters are the same as the ResNet50 network, and the compression rate in the SE module is set to 1/8.

### Performance evaluation and statistical analysis

Mean absolute error (MAE), mean absolute percentage error (MAPE), and coefficient value (R^2^) of refractive prediction were used to assess the predictive performance in the testing group, which was calculated using the following equation, where *n* is the sample size, $y = \left\{ {y_{1} ,y_{2} , \ldots ,y_{n} } \right\}$ is the actual value, and $\hat{y} = \left\{ {\hat{y}_{1} ,\hat{y}_{2} , \ldots ,\hat{y}_{n} } \right\}$ is the predicted value, $\overline{y}$ is the average value. A nonparametric method for the Bland–Altman plot analysis was used to evaluate the relationship between predictive and actual Hb loss, wherein bias and limits of agreement (LOA) were calculated^24^.

$$\begin{aligned} MAE & = \frac{1}{n}\mathop \sum \limits_{i = 1}^{n} \left| {\hat{y}_{i} - y_{i} } \right| \\ MAPE & = \frac{100\% }{n}\mathop \sum \limits_{i = 1}^{n} \left| {\frac{{\hat{y}_{i} - y_{i} }}{{y_{i} }}} \right| \\ R^{2} & = 1 - \frac{{\mathop \sum \nolimits_{i = 1}^{n} \left( {y_{i} - \hat{y}_{i} } \right)^{2} }}{{\mathop \sum \nolimits_{i = 1}^{n} \left( {y_{i} - \overline{y}} \right)^{2} }} \\ \end{aligned}$$

## Result

A total of 595 images were included in the training group to develop the surgical Hb loss prediction model, and 256 images were included in the testing group to evaluate the model performance. In the present study, the deep learning model based on the SE-ResNet50 network was selected as the optimal surgical Hb loss prediction model owing to the best performance and the smallest bias with the narrowest LOA. As shown in Table 1, the MAE, MAPE, and R^2^ values for the method based on the feature extraction model were 27.34 mg, 21.9%, and 0. 96, respectively. The ResNet-50 network presented higher R^2^ values (0.99) and lower MAE (14.66 mg) and MAPE values (10.7%), which were more satisfactory than those of the methods based on the feature extraction model. However, the deep learning model based on the SE-ResNet50 network was presented with 11.09 mg of MAE and 8.6% of MAPE, and the Hb predictive values were highly correlated with the Hb actual reference value (R^2^ = 0.99), which were more satisfactory than those of the methods based on feature extraction model and ResNet-50 network.

**Table 1:** Model performances of surgical hemoglobin loss estimation.

Algorithms	MAE, mg	MAPE (%)	R^2^ score
Feature Extraction	27.34	21.9	0.96
ResNet-50	14.66	10.7	0.99
SE-ResNet50	11.09	8.6	0.99

**Figure 4:**
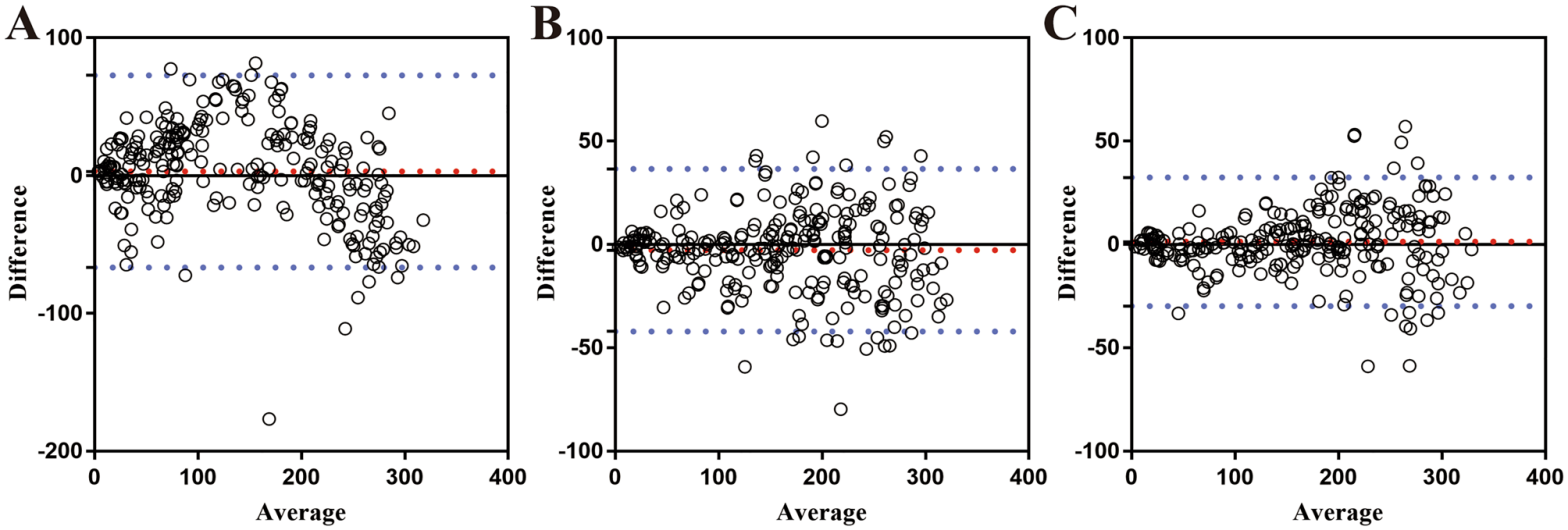
Bland–Altman plots analysis of the concordance between predictive and actual Hb loss. (**A**) Bland–Altman plots analysis based on feature extraction technology (**B**) Bland–Altman plots analysis based on ResNet-50 (**C**) Bland–Altman plots analysis based on SE-ResNet50. The dashed red line represents the bias (mean difference) and the dashed blue lines represent the upper and the lower limits of agreement.

The Bland–Altman plot analysis of three predictive models was applied to evaluate the concordance between the Hb predictive value based on the different predictive models and the actual Hb losses, which are shown in Fig. 4. The biases of the methods based on the feature extraction model and ResNet-50 network were 3.013 mg (95% CI − 1.34 mg, 7.37 mg) and − 2.74 mg (95% CI − 5.2 mg, − 0.28 mg), the corresponding lower LOA were − 66.68 mg (95% CI − 71.04 mg, − 62.32 mg) and − 42.09 mg (95% CI − 44.54 mg, − 39.63 mg), the corresponding upper LOA were 72.7 mg (95% CI 68.34 mg, 77.06 mg) and 36.62 mg (95% CI 34.16 mg, 39.08 mg), respectively. The Bland–Altman plot analysis of the SE-ResNet50 network revealed the smallest bias (1.343 mg; 95% CI − 0.6, 3.29 mg) with the narrowest LOA, the corresponding lower LOA was − 29.81 mg (95% CI − 31.76, − 27.86 mg) and the upper LOA was 32.5 mg (95% CI 30.55, 34.45 mg). The comparisons of the bias value with the corresponding limit of agreement between these predictive model values and actual value are shown in Table 2. In addition, to further evaluate real-time capabilities for surgical hemoglobin loss estimation, the average computing time required to calculate hemoglobin loss per image was calculated. As shown in Table 3, an average of 37 and 36 images per second can be processed to calculate surgical hemoglobin loss for the ResNet-50 and SE-ResNet50 networks, respectively, which can meet the requirements of real-time processing. Therefore, the deep-learning model based on the SE-ResNet50 network was the optimal predictive model for real-time and accurate estimation of surgical Hb loss.

**Table 2:** The concordance between the model hemoglobin loss predictive value and actual value.

Algorithms	Bias (95% CI), mg	Lower LOA (95% CI), mg	Upper LOA (95% CI), mg
Feature extraction	3.013 (− 1.34, 7.37)	− 66.68 (− 71.04, − 62.32)	72.7 (68.34, 77.06)
ResNet-50	− 2.74 (− 5.2, − 0.28)	− 42.09 (− 44.55, 39.63)	36.62 (34.16, 39.08)
SE-ResNet50	1.343 (− 0.6, 3.29)	− 29.81 (− 31.76, − 27.86)	32.5 (30.55, 34.45)

**Table 3:** Average computing time required to calculate hemoglobin loss per image.

Algorithms	Average computing time per image (s)
Feature extraction	0.0438
ResNet-50	0.0268
SE-ResNet50	0.0274

### Model visualization

**Figure 5:**
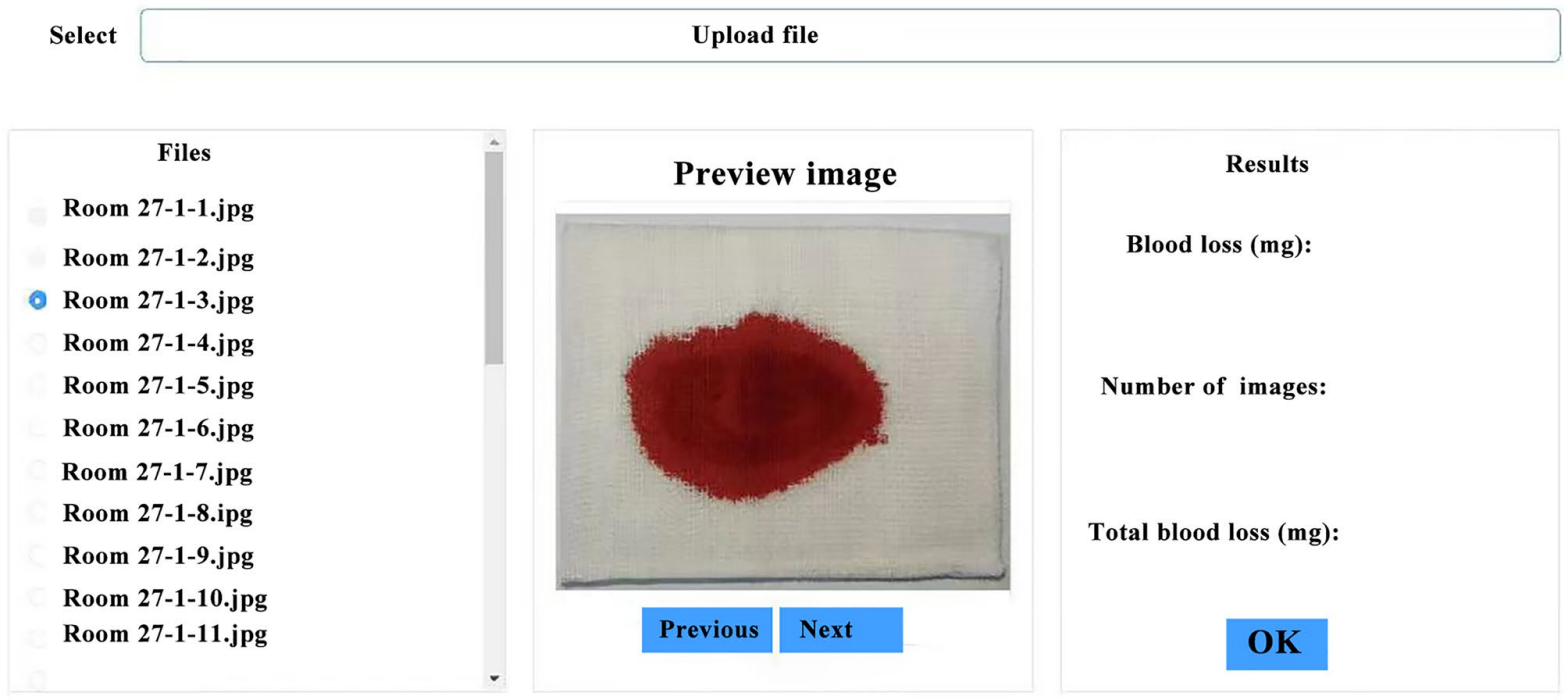
Interactive interface for surgical Hb loss estimation provided by the deep learning model based on SE-ResNet50.

We have designed an interactive interface to more intuitively display surgical Hb loss provided by the optimal deep learning model based on the SE-ResNet50 network, which is shown in Fig. 5. The interface includes a user upload area on the left, an image previews area in the middle, and a surgical Hb loss prediction area on the right. In the prediction view, the circulating nurse or surgeons can upload an unlimited number of intraoperative blood-soaked sponge images in the user upload area and click the surgical Hb loss prediction button to see the cumulative number of uploaded medical sponge images and total surgical Hb loss prediction. This interactive interface helps surgeons and anesthesiologists in real-time estimate surgical Hb loss.

## Discussion

In recent years, with the rapid development of minimally invasive surgery and the continuous expansion of the application area of robotic surgery, surgical blood loss has also become increasingly low. Intraoperative blood loss of no more than 50 ml occurs in many major surgeries^25–27^. However, it remains an important indicator to assess the technical quality of the surgery. Therefore, real-time estimation of surgical blood loss, even Hb loss, is urgently needed in clinical practice, especially in the technical evaluation of surgical quality. In the present study, we developed three predictive models (Feature extraction technology, ResNet-50, and SE ResNet50) to predict intraoperative Hb loss as a more accurate alternative method to estimate perioperative blood loss. By comparing the predictive performance and the concordance between the Hb predictive value and the actual Hb value of three predictive models. We concluded that the deep-learning model based on the SE-ResNet50 network is the optimal model for real-time and accurate estimation of surgical Hb loss owing to the best predictive performance and the smallest bias with the narrowest LOA. Besides, we also designed an interactive interface to further achieve real-time and accurate automatic calculation of surgical Hb loss.

So far, numerous studies have proposed various methods for surgical blood loss estimation on surgical sponges. First, visual estimation was the most common measurement method for blood loss estimation, which has been proven notoriously inconsistent and inaccurate^8^. Although many technical methods were used to improve the accuracy of visual estimation, including simulated clinical reconstruction and didactic training, these depend on the experience level of surgeons, and the blood loss estimation skill easily declined after several months of didactic training^28,29^. Besides, gravimetric estimation is considered relatively accurate for estimating surgical blood loss. Still, it was easy to overestimate blood loss due to contaminants other than blood on the surgical sponges, and it is also impractical due to being time-consuming and labor-intensive^8,9^. Recently, a new monitoring platform (Triton System, Gauss Surgical, Inc., Los Altos, USA) based on the feature extraction technology has been proposed, a camera-enabled mobile application that allows intraoperative scanning of surgical sponges to measure Hb mass directly^30,31^. Still, the detailed algorithm for Hb loss estimation is being determined, and the accuracy of this system also remains to be further increased. Moreover, all the blood-soaked sponges in previous studies were from medical waste, the actual mass blood and Hb loss were unknown, and the indicators for model performance evaluation, such as MAE, MAPE, and R^2^, were not shown. Therefore, the accuracy assessment for these studies could have been more reliable.

Currently, artificial intelligence technology is increasingly applied in the medical field. For instance, as a subdiscipline of artificial intelligence, deep learning has been developed to conduct all kinds of work involved in medical image processing and analysis. A recent study also developed a novel method for Hb loss estimation based on feature extraction technology and deep learning methods using the blood-soaked sponge, and they believed the relationship between the extracted feature parameters and surgical Hb loss may be non-linear and even more complex. They also confirmed that the deep learning method based on DenseNet was more accurate than those based on linear regression of feature extraction technology, random forest (RF), and extreme gradient boosting (Xgboost). Still, the R^2^ and MAE for Hb loss estimation based on the DenseNet model were 0.941 (95% CI 0.934–0.948) and 0.325 (95% CI 0.293–0.355), Bland–Altman analysis revealed a bias of 0.05 g with narrow LOA (− 0.87 to 0.97 g) between the methods based on DenseNet and actual blood loss and Hb loss^32^. Although the model performance and concordance based on DenseNet were better than those based on the above models, it was not as good as that of the SE-ResNet50 model we proposed. In the present study, we developed three predictive models to obtain the best surgical blood loss prediction, which included a linear regression model based on image feature extraction technology, and deep learning models based on ResNet-50 and SE-ResNet50. Finally, the deep learning methods based on SE-ResNet50 achieved the best predictive performance and the smallest bias with the narrowest LOA. The MAE, MAPE, and R^2^ for the SE-ResNet50 model were 11.09 mg, 8.6%, and 0.99, respectively. The biases and limits of the agreement were 1.343 mg and − 29.81 mg to 32.5 mg between predictive Hb loss of the methods based on SE-ResNet50 and actual Hb loss.

The linear regression models based on image feature extraction technology may have shortcomings in predicting surgical Hb loss. As mentioned in the above literature, the relationship between the extracted feature parameters and surgical Hb loss may be non-linear and even more complex. Therefore, we developed the deep learning model based on ResNet-50, and MAE, MAPE, and R^2^ values improved by 12.68 mg, 11.2%, and 0.03 compared to feature extraction technology. However, to further improve the performance of the model, SE blocks were added to the backbone network ResNet-50 to produce the SE-ResNet50 module. SE block was one of the channel attention modules, which mainly included Squeeze and Excitation blocks. The Squeeze blocks mainly compress the spatial information of the input feature image, while the Excitation blocks combine the channel attention information with the input feature image to ultimately obtain a featured image with channel attention. In the present study, The deep learning model based on the SE-ResNet50 module has effectively improved its model performance and bias degree with LOA. Compared with the deep learning model based on ResNet-50, the SE-ResNet50 model has an improvement of 3.57 mg and 2.1% in MAE and MAPE values, respectively, besides, the concordance based on the SE-ResNet50 module was a smaller bias with the narrower LOA than those based on the other two predictive models. Therefore it has been the optimal model for surgical Hb loss prediction owing to the best predictive performance and the smallest bias with the narrowest LOA. Certainly, the deep learning model based on the SE-ResNet module has also been widely applied in clinical practice and has also achieved good results. Jiang et.al designed a novel convolutional neural network based on a small SE-ResNet module, which was used for the automatic classification of breast cancer histology images into benign and malignant and eight subtypes, and achieved satisfactory accuracy for the binary classification (98.87% to 99.34%) and the multi-class classification (90.66% to 93.81%)^33^. Yin et.al also developed two deep learning models (SE_CT_ and SE_PET_) with SE-ResNet module for the prediction of epidermal growth factor receptor (EGFR) mutation with CT and PET images, respectively. The AUC was further improved to 0.84 after integrating SE_CT_ and SE_PET_ with stacked generalization, which is capable to predict the EGFR mutation status of patients with lung adenocarcinoma automatically and non-invasively^34^. Hu et.al also developed the SE-ResNet50-based chemotherapy response prediction system from pretreatment CT images preprocessed with an imaging oversampling method, and then the deep learning signature and clinic-based features were fed into the deep learning radio-clinical signature, which accurately predicts tumor response and identifies the risk of overall survival in locally advanced gastric cancer patient priors to neoadjuvant chemotherapy^35^. In addition, we have designed an interactive interface to further realize real-time and accurate surgical Hb loss. The circulating nurse could take photos of these blood-soaked sponges and upload them to this interactive interface, and surgical Hb loss would be automatically calculated in real-time.

However, some limitations should be acknowledged in this study. First, just one kind of sponge was used in this study. Second, the sample of images with blood-soaked sponges might not be large enough for typical deep learning methods, although it also achieved a satisfactory performance. Therefore, further studies with large samples of other sizes of sponges and canisters should be performed.

## Conclusion

It is feasible for real-time and accurate surgical Hb loss estimation using deep learning-based medical sponges image analysis, especially for laparoscopic and robotic surgeries. Objective and precise assessment of surgical Hb loss was helpful for clinical decisions and technical evaluation.

## Data Availability

The datasets generated during and/or analyzed during the current study are available from the corresponding author on request.
